# How Salty Are Your Fluids? Pediatric Maintenance IV Fluid Prescribing Practices Among Hospitalists

**DOI:** 10.3389/fped.2019.00549

**Published:** 2020-01-15

**Authors:** Alan M. Hall, Juan C. Ayus, Michael L. Moritz

**Affiliations:** ^1^Division of Hospital Medicine and Pediatrics, University of Kentucky College of Medicine, Lexington, KY, United States; ^2^Renal Consultants of Houston, Houston, TX, United States; ^3^School of Medicine, University of California, Irvine, Irvine, CA, United States; ^4^Department of Pediatrics, UPMC Children’s Hospital of Pittsburgh, University of Pittsburgh School of Medicine, Pittsburgh, PA, United States

**Keywords:** IV fluids, maintenance, pediatric hospitalists, hyponatremia, IV fluid therapy

## Abstract

**Objective:** The primary goal of this study was to assess current maintenance intravenous fluid (mIVF) prescribing practices of pediatric hospitalists after the release of the American Academy of Pediatrics Clinical Practice Guideline (AAP CPG), specifically assessing the rates of various isotonic vs. hypotonic solutions used in discrete age groups and in common clinical scenarios associated with anti-diuretic hormone (ADH) excess and hyponatremia. We hypothesized that isotonic fluids would be selected in most cases outside of the neonatal period.

**Methods:** A voluntary and anonymous survey was distributed to the LISTSERV® for the AAP Section on Hospital Medicine.

**Results:** There were 402 total responses (10.1% response rate) with the majority of respondents being pediatric hospitalists. Isotonic solutions were preferred by respondents in older children compared to younger age groups, at 87.8% for the 1–18 years age group compared to 66.3% for the 28 days to 1 year age group and 10.6% for the younger than 28 days age group (all *p* values <0.0001). When presented with disease states associated with ADH excess, isotonic fluids were preferred in higher percentages in all age groups except in children younger than 28 days when 0.45% sodium chloride was preferred; 0.2% sodium chloride was rarely chosen.

**Conclusions:** Overall, based on survey responses, pediatric hospitalists are following the 2018 AAP CPG on mIVF and are more likely to choose isotonic fluids as their primary mIVF in pediatric patients outside of the neonatal period, including in scenarios of excess ADH. Isotonic fluids use seems to be higher with increasing age and hypotonic fluids are more commonly chosen in the neonatal period.

## Introduction

Maintenance intravenous fluids (mIVF) are currently a popular topic among pediatric hospitalists. Risk factors for excess secretion of anti-diuretic hormone (ADH) have been increasingly described, placing hospitalized children at risk for hyponatremia (1). Numerous randomized-controlled trials led to several meta-analyses and reviews showing a decreased risk of hyponatremia with isotonic fluids compared to hypotonic fluids in hospitalized children (2–4). As a result, the 2015 National Institute for Health and Care Excellence guideline and the 2018 American Academy of Pediatrics Clinical Practice Guideline (AAP CPG) both recommend isotonic mIVF for most children (5, 6).

Several published surveys have assessed prescribing practices for mIVF in children with high rates of hypotonic fluids selected. Way et al. surveyed anesthesiologists in the United Kingdom in 2006 and showed that most chose a hypotonic fluid post-operatively (7). Davies et al. published similar results after surveying anesthesiologists and pediatric surgeons in 2008, also in the United Kingdom (8). Keijzers et al. surveyed a wide-range of physicians at an Australian hospital in 2012 and found that most prescribed hypotonic fluids in a clinical scenario with high risk for excess ADH secretion (9). Also in 2012, Freeman et al. showed that pediatric residents in the United States were more likely to choose hypotonic mIVF (10). Lee et al. published similar survey results for pediatric residents in South Korea in 2013 (11).

No survey has focused on pediatric hospitalists and no survey has been published since the release of the AAP CPG. Our primary goal of this survey was to assess current mIVF prescribing practices among pediatric hospitalists after the release of this guideline, specifically assessing the prescribing rates of isotonic vs. hypotonic fluids. We hypothesized that isotonic fluids would be selected in most cases outside of the neonatal period. As a secondary objective, we sought to assess if hospital practice or year of training completion influenced mIVF choices.

## Methods

A voluntary and anonymous survey using Research Electronic Data Capture was distributed to the LISTSERV® for the AAP Section on Hospital Medicine (12). This LISTSERV® exists for providers in pediatric hospital medicine but also welcomes trainees and other providers considering a career change to hospital medicine. As of March 18, 2019, there were 3,964 emails registered on the LISTSERV®. The survey was emailed on March 25, 2019 and was open for 28 days with an additional email reminder sent at day 14. The survey was developed iteratively using a previously published survey as a guide (10).

In the survey, respondents were asked to provide demographic data including their primary job/specialty, hospital practice category, and period of residency completion. Next, they were asked their primary fluid choice for children younger than 28 days, 28 days to 1 year, and 1–18 years of age. Prior to data analysis, it was decided that isotonic solutions would include 0.9% sodium chloride, Ringer's lactate, and Plasma-Lyte 148. Hypotonic solutions would include 0.2% sodium chloride and 0.45% sodium chloride. Finally, respondents were presented four clinical scenarios and asked to pick their mIVF choice if the patient was a 27 day-old neonate, a 6 month-old infant, or a 13 year-old adolescent (if applicable) for each scenario (gastroenteritis, meningitis, bronchiolitis, and post-surgical from a Nissen fundoplication). In the clinical scenarios, the respondents were asked to assume that dextrose and potassium would be added to the fluids as needed and the patients were: (1) average weight for age, (2) euvolemic with adequate urine output after 1–2 boluses, if needed, (3) without electrolyte disturbances, and (4) unable to tolerate enteral fluids. See Supplementary Material for the full survey.

Statistical analysis was performed using the chi-squared test to assess differences between specific groups. The primary author's institutional review board approved this study.

## Results

**Figure 1:**
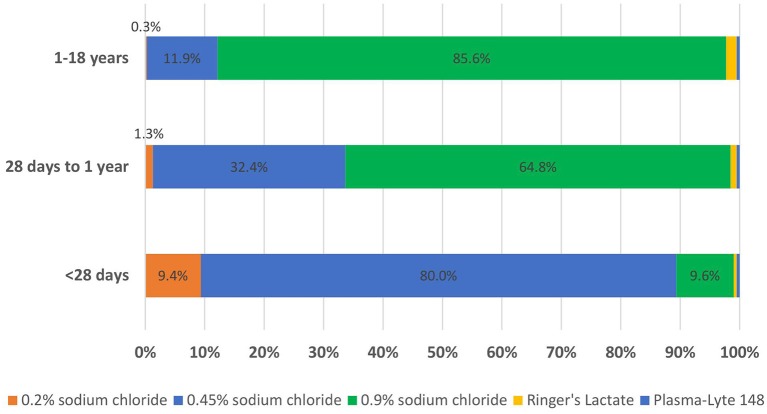
Primary maintenance intravenous fluid, by age.

The demographic data of the respondents are detailed in Table 1 with 402 overall respondents resulting in a 10.1% response rate. Figure 1 displays the primary mIVF chosen for each age range, with 0.9% sodium chloride and 0.45% sodium chloride the two most commonly chosen solutions (53.3 and 41.4%, respectively). Isotonic solutions were preferred by respondents in older children compared to younger age groups, at 87.8% for the 1–18 years age group compared to 66.3% for the 28 days to 1 year age group and 10.6% for the younger than 28 days age group (all *p* values < 0.0001). 0.45% saline was the preferred mIVF in the younger than 28 days age group (80.0%).

**Table 1:** Response rate and demographics.

**Survey response**	**Total**	**Percentage (%)**
Survey response rate	402/3,964	10.1
**Primary job/Specialty**		
Pediatric hospitalist	317	78.9
Pediatric hospitalist, fellowship trained	30	7.5
Med-Peds hospitalist	13	3.2
Resident or fellow in pediatric hospital medicine	23	5.7
Other	19	4.7
**Hospital practice**		
Free-standing children's hospital	151	37.6
Children's hospital within an adult hospital	151	37.6
Primarily adult hospital that admits children	100	24.9
**Period completed residency**		
Prior to 1990	24	6.0
1990–1999	35	8.7
2000–2009	98	24.4
2010 or later	232	57.7
I have not completed a pediatric residency	13	3.2

**Figure 2:**
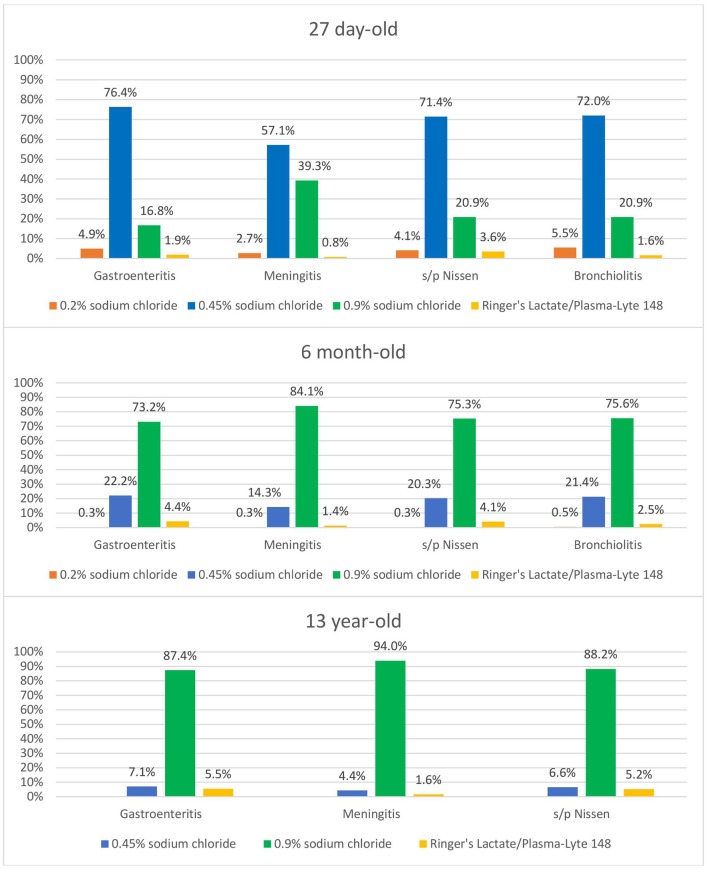
Maintenance IV fluids selected by indication, for each age group.

Comparing the primary mIVF chosen for each age range by hospital practice location, isotonic fluids were more likely to be used in children younger than 28 days old in children's hospitals (free-standing or within an adult hospital) at 13.1% compared to non-children's hospitals at 3.1% (*p* = 0.0055). The influence of time of training completion on primary mIVF chosen, demonstrated a statistical difference only in patients 1–18 years of age, with residency training completed in 2010 or later showing a higher rate of an isotonic fluid choice (90.7%) compared to those who completed training prior to 2010 (83.3%, *p* = 0.0298). When presented with specific scenarios associated with ADH excess, the overall use of isotonic fluids increased to 64.4%, with higher usage with increasing age in the three groups (27 day-old, 6-month old, and 13 year-old; see Figure 2). For the 13 year-old, >87% of respondents chose isotonic fluids for each scenario, without statistical differences between the cases. For the other two age ranges, isotonic fluids were more likely to be chosen in the meningitis case compared to the other three cases (*p* < 0. 00001 for 27 day-old and *p* < 0.034 in 6 month-old).

When the scenarios were divided by time of training completion, response rates were higher for isotonic fluids in those who finished training in 2010 or later compared to those who completed their training prior to 2010 for the following cases: 6 month-old with gastroenteritis (82.4% vs. 70.3%, *p* = 0.0076) and bronchiolitis (83.3% vs. 70.3%, *p* = 0.0037) and 13 year-old with meningitis (97.6% vs. 92.4%, *p* = 0.0193). The opposite was true in the post-surgical case for the 27 day-old when those who completed training prior to 2010 were more likely to choose isotonic fluids (32.6%) compared to those who completed training after 2010 (19.5%, *p* = 0.005).

## Discussion

The majority of pediatric hospitalists surveyed chose isotonic mIVF for children 28 days to 18 years of age, which is consistent with the 2018 AAP CPG and vastly different from the preference for hypotonic fluids in previously published pediatric surveys (6–11). The AAP CPG specifically excluded neonates in their recommendation to use isotonic fluids given the paucity of data for this age group; our results show that hypotonic fluids continue to be more commonly chosen for neonates younger than 28 days (6).

While hypotonic fluids remain the preferred solution in neonates, 0.45% sodium chloride was favored compared to 0.2% sodium chloride, which is the closest solution in sodium concentration to the requirements in the original 1957 publication by Holliday and Segar that laid the foundation for mIVF prescribing practices in children (13). This potentially suggests that the AAP CPG and supporting literature may have influenced fluid choice in neonates despite the absence of a specific recommendation for this age group. Outside of the neonatal period, isotonic fluids were the most common choice in all clinical scenarios presented. Each clinical scenario was chosen to represent a condition commonly associated with excess ADH making isotonic fluids the preferred choice.

We observed higher rates of isotonic fluids in specific scenarios in children's hospitals compared to non-children's hospitals and in those who completed residency training in 2010 or later compared to training prior to 2010. However, the opposite was observed in the 27-day-old post-surgical case, when isotonic fluids were more common in those who completed their training prior to 2010. In certain scenarios, pediatric hospitalists may be more likely to prescribe isotonic fluids if they have more recently completed their residency training or if they practice in a children's hospital. Both of these factors may increase the likelihood of exposure to the recent literature describing the decreased risk of hyponatremia with isotonic fluids.

Balanced isotonic solutions (Ringer's lactate and Plasma-Lyte 148) were uncommonly chosen in any scenario and both had much lower rates compared to 0.9% sodium chloride. Though there is limited adult data to support the use of balanced isotonic fluids, there is scarce data on this topic for mIVF in pediatrics, which we suspect contributed to these low rates (14, 15). Limitations of this study include that this survey data may not accurately predict prescribing behavior and may not be a representative sample of pediatric hospitalists in the United States. A participation bias is also possible as survey respondents may have been more aware of the AAP CPG and preferentially more likely to choose isotonic fluids. Additionally, the LISTSERV® included other respondents, who were not primary hospitalists, which may have influenced the results.

## Conclusion

Overall, based on survey responses, pediatric hospitalists are following the 2018 AAP CPG on mIVF and are more likely to choose isotonic fluids as their primary mIVF in pediatric patients outside of the neonatal period, including in scenarios of excess ADH. Isotonic fluids use seems to be higher with increasing age and hypotonic fluids are more commonly chosen in the neonatal period. What remains to be determined from the change in mIVF from hypotonic to isotonic fluid is if it will result in unintended consequences, such as hypernatremia, and how often electrolytes need to be monitored. An ongoing multicenter quality improvement project will help address this issue.

## Data Availability Statement

The datasets generated for this study are available on request to the corresponding author.

## Author Contributions

AH and MM contributed conception and design of the study. AH collected and organized the data and wrote the first draft of the manuscript. All authors contributed to data analysis, interpretation, manuscript revision, read, and approved the submitted version.

### Conflict of Interest

The authors declare that the research was conducted in the absence of any commercial or financial relationships that could be construed as a potential conflict of interest.
