# Supplementary material for: How Salty Are Your Fluids? Pediatric Maintenance IV Fluid Prescribing Practices Among Hospitalists
Source: Front Pediatr. 2020 Jan 15;7:549. doi: 10.3389/fped.2019.00549 (PMC6974532; doi:10.3389/fped.2019.00549)
Supplement: Supplementary file 1 [file Data_Sheet_1.PDF]

# Pediatric Maintenance IV Fluid Survey

Dear Colleague:

A researcher at the University of Kentucky is inviting you to take part in a survey about maintenance IV fluid prescribing practices. This study aims to gather data on current maintenance IV fluid prescribing practices.

We will be sending out this survey via email to members of the AAP Section on Hospital Medicine Listserv. This survey will be emailed a total of two times over a period of two weeks via RedCap to garner as many responses as possible. As we will not know who has already completed the study in the interest of preserving confidentiality, you will receive two separate e-mail promptings to complete the survey. You need only to complete the survey once.

Although you may not get personal benefit from taking part in this research study, your responses may help us understand more about maintenance IV fluid prescribing practices. Some volunteers experience satisfaction from knowing they have contributed to research that may possibly benefit others in the future.

The survey will take less than 5 minutes to complete.

There are no known risks to participating in this study other than a potential breach of confidentiality. However, we will make every effort to minimize this risk.

Your response to the survey will be kept confidential to the extent allowed by law. When we write about the study you will not be identified.

We hope to receive completed questionnaires from about 200 people, so your answers are important to us. Of course, you have a choice about whether or not to complete the survey, but if you do participate, you are free to skip any questions or discontinue at any time.

Please be aware, while we make every effort to safeguard your data once received on our servers via REDCap, given the nature of online surveys, as with anything involving the Internet, we can never guarantee the confidentiality of the data while still en route to us.

If you have questions about the study, please feel free to ask; my contact information is given below. If you have complaints, suggestions, or questions about your rights as a research volunteer, contact the staff in the University of Kentucky Office of Research Integrity at 859-257-9428 or toll-free at 1-866-400-9428.

Thank you in advance for your assistance with this important project.

Sincerely,

Alan Hall, MD
Assistant Professor
Department of Internal Medicine & Pediatrics
College of Medicine

University of Kentucky
Phone: 859-562-2109
E-mail: alan.hall@uky.edu

| | |
|---|---|
| 1. Please describe your primary job/specialty | ◯ Pediatric Hospitalist, fellowship trained in pediatric hospital medicine<br>◯ Pediatric Hospitalist, not fellowship trained in pediatric hospital medicine<br>◯ Med-Peds Hospitalist<br>◯ Outpatient Pediatrician<br>◯ Pediatric Intensivist<br>◯ Other Pediatric Subspecialist<br>◯ Fellow in Pediatric Hospital Medicine<br>◯ Resident in Pediatrics<br>◯ Medical Student<br>◯ Advanced Practice Provider (Physician Assistant or Nurse Practitioner)<br>◯ Other |
| Please describe | ________________________________ |
| 2. Please select the hospital category where you currently practice pediatrics. | ◯ Free-standing children's hospital<br>◯ Children's hospital within an adult hospital<br>◯ Primarily adult hospital that will admit children |
| 3. Please select the period that you completed your pediatric residency | ◯ Prior to 1990<br>◯ 1990 - 1999<br>◯ 2000 - 2009<br>◯ 2010 or later<br>◯ I have not completed a pediatric residency |

| | |
|---|---|
| 4. Which is your primary maintenance intravenous fluid selection for patients younger than 28 days of age admitted to your service? Assume that appropriate dextrose and potassium chloride will be added to the intravenous fluids. | ◯ 0.2% sodium chloride<br>◯ 0.45% sodium chloride<br>◯ 0.9% sodium chloride (normal saline)<br>◯ Ringer's Lactate<br>◯ Plasma-Lyte 148 |
| 5. Which is your primary maintenance intravenous fluid selection for patients 28 days to 1 year of age admitted to your service? Assume that appropriate dextrose and potassium chloride will be added to the intravenous fluids. | ◯ 0.2% sodium chloride<br>◯ 0.45% sodium chloride<br>◯ 0.9% sodium chloride (normal saline)<br>◯ Ringer's Lactate<br>◯ Plasma-Lyte 148 |
| 6. Which is your primary maintenance intravenous fluid selection for patients 1 year to 18 years of age admitted to your service? Assume that appropriate dextrose and potassium chloride will be added to the intravenous fluids. | ◯ 0.2% sodium chloride<br>◯ 0.45% sodium chloride<br>◯ 0.9% sodium chloride (normal saline)<br>◯ Ringer's Lactate<br>◯ Plasma-Lyte 148 |

**Please choose the appropriate maintenance fluid for the following clinical scenarios. Assume that the patient:**

- **Is of average weight for age**
- **Is euvolemic with adequate urine output after 1-2 boluses, if needed**
- **Has no electrolyte disturbances**
- **Is unable to tolerate oral or tube feeds**
- **Will have dextrose and potassium added as needed to the fluid**

7. A patient is admitted to your service who presents with vomiting and diarrhea consistent with viral gastroenteritis. The patient's diarrhea has now resolved, but he continues to experience frequent emesis.

| | 0.2% sodium chloride | 0.45% sodium chloride | 0.9% sodium chloride (normal saline) | Ringer's Lactate | Plasma-Lyte 148 |
|---|---|---|---|---|---|
| 27 day-old neonate | ○ | ○ | ○ | ○ | ○ |
| 6 month-old infant | ○ | ○ | ○ | ○ | ○ |
| 13 year-old adolescent | ○ | ○ | ○ | ○ | ○ |

8. A patient is admitted to your service due to a clinical presentation and initial LP findings concerning for meningitis. The patient is irritable and mildly somnolent.

| | 0.2% sodium chloride | 0.45% sodium chloride | 0.9% sodium chloride (normal saline) | Ringer's Lactate | Plasma-Lyte 148 |
|---|---|---|---|---|---|
| 27 day-old neonate | ○ | ○ | ○ | ○ | ○ |
| 6 month-old infant | ○ | ○ | ○ | ○ | ○ |
| 13 year-old adolescent | ○ | ○ | ○ | ○ | ○ |

9. A patient who has just undergone a Nissen fundoplication for severe reflux is transferred to your service from the surgery team with instructions to keep the patient NPO for 48 hours. The patient will be maintained on a morphine PCA pump during this 48 hour period.

| | 0.2% sodium chloride | 0.45% sodium chloride | 0.9% sodium chloride (normal saline) | Ringer's Lactate | Plasma-Lyte 148 |
|---|---|---|---|---|---|
| 27 day-old neonate | ○ | ○ | ○ | ○ | ○ |
| 6 month-old infant | ○ | ○ | ○ | ○ | ○ |
| 13 year-old adolescent | ○ | ○ | ○ | ○ | ○ |

10. A patient is admitted to your service in moderate respiratory distress with a diagnosis of acute viral bronchiolitis.

| | 0.2% sodium chloride | 0.45% sodium chloride | 0.9% sodium chloride (normal saline) | Ringer's Lactate | Plasma-Lyte 148 |
|---|---|---|---|---|---|
| 27 day-old neonate | ○ | ○ | ○ | ○ | ○ |
| 6 month-old infant | ○ | ○ | ○ | ○ | ○ |
